# McMAC: Towards a MAC Protocol with Multi-Constrained QoS Provisioning for Diverse Traffic in Wireless Body Area Networks

**DOI:** 10.3390/s121115599

**Published:** 2012-11-12

**Authors:** Muhammad Mostafa Monowar, Mohammad Mehedi Hassan, Fuad Bajaber, Musaed Al-Hussein, Atif Alamri

**Affiliations:** 1 Department of Information Technology, Faculty of Computing and Information Technology, King AbdulAziz University, 21589 Jeddah, Saudi Arabia; E-Mail: fbajaber@kau.edu.sa; 2 College of Computer and Information Sciences, King Saud University, 11543 Riyadh, Saudi Arabia; E-Mails: mmhassan@ksu.edu.sa (M.M.H.); musaed@ksu.edu.sa (M.A.-H.); atif@ksu.edu.sa (A.A.)

**Keywords:** QoS, Wireless Body Area Networks, heterogeneous traffic

## Abstract

The emergence of heterogeneous applications with diverse requirements for resource-constrained Wireless Body Area Networks (WBANs) poses significant challenges for provisioning Quality of Service (QoS) with multi-constraints (delay and reliability) while preserving energy efficiency. To address such challenges, this paper proposes McMAC, a MAC protocol with multi-constrained QoS provisioning for diverse traffic classes in WBANs. McMAC classifies traffic based on their multi-constrained QoS demands and introduces a novel superframe structure based on the “transmit-whenever-appropriate” principle, which allows diverse periods for diverse traffic classes according to their respective QoS requirements. Furthermore, a novel emergency packet handling mechanism is proposed to ensure packet delivery with the least possible delay and the highest reliability. McMAC is also modeled analytically, and extensive simulations were performed to evaluate its performance. The results reveal that McMAC achieves the desired delay and reliability guarantee according to the requirements of a particular traffic class while achieving energy efficiency.

## 1. Introduction

The massive proliferation of Micro-Electro-Mechanical Systems (MEMS) [1] as well as the wide adoption of wireless networking technologies have created significant opportunities for the wide-spread utilization of various innovative applications in the foreseeable future. One of the most striking application domains receiving growing interest is health care. A Wireless Body Area Network (WBAN) is one such recent technology for fostering health care applications that integrate intelligent, miniaturized, and low-power sensor nodes in/around the human body to monitor body functions and the surrounding environment.

Initially developed with vital sign monitoring applications, WBANs nowadays facilitate a wider range of applications, including interactive body computing, education, and entertainment [2,3]. The advent of such diverse applications thus necessitates designing an effective and generic [4] communication protocol for WBAN that could support the distinct nature of heterogeneous applications.

Due to the energy constraint of battery-driven body sensor nodes, energy conservation is given primary concern when designing the communication protocols for WBANs. A good number of proposals focusing on energy-efficient MAC layer solutions already exist in the literature [5–17]. These protocols are mainly aimed at optimizing the sleep duration of the nodes, maintaining a low duty cycle that extends the lifetime of the network. In focusing more on energy conservation, however, these protocols overlooked the QoS requirements of diverse applications, which is no less important than providing energy efficiency. If the QoS requirements are not met properly, the objective of the application becomes futile no matter how long the lifetime of the network can last.

Considering the diverse application requirements of a WBAN, the most important QoS metrics, along with energy efficiency, are *delay* and *reliability*, which we denote together as *multi-constrained QoS*. However, not all applications have similar requirements in satisfying the multi-constrained QoS. For instance, electrocardiogram (ECG) traffic has strict delay and reliability requirements, whereas the respiration monitoring application possesses only a reliability constraint and may tolerate latency. Moreover, it is also more likely that a single WBAN would consist of multiple body sensor nodes with diverse QoS requirements. Therefore, the existence of such a heterogeneous traffic environment in the same network requires the treatment of each traffic class based on its respective multi-constrained QoS demands, not only for the preservation of the application objective but also for the proper utilization of the limited resources of WBANs.

Although most of the works on MAC protocols for WBANs focused on energy-efficiency, several works [18–25] promoted QoS provisioning issues. However, these proposals mainly concentrated on satisfying the QoS requirements based on traffic priority only, which might not reflect the actual multi-constrained QoS requirements and thus could cause overestimation in allocating resources for the resource-constrained WBAN. Hence, to the best of our knowledge, the issue of dealing with each traffic class according to its appropriate multi-constrained QoS demands without sacrificing energy efficiency remains to be addressed.

In this paper, we aim to provide such a generic solution, which, however, faces several challenges. First, providing energy efficiency requires the body sensor nodes to be in periodic sleep state. However, keeping all the nodes awake during the active period causes unnecessary idle listening and overhearing, resulting in waste of energy. Second, allowing diverse nodes to participate in the same active period results in high contention and thereby collision, which reduces reliability for the loss-intolerant traffic to a greater extent. Third, ensuring the highest reliability for the loss-intolerant traffic requires a guaranteed and collision-free slot. The allocation of such slots demands additional resources. Because not all types of traffic require such resources, allocating a collision-free slot independent of traffic type wastes resource for the resource-constrained WBAN. Finally, one of the important consideration for a WBAN is an efficient emergency traffic handling mechanism. Emergency traffic is sporadic in nature and needs to be delivered instantaneously with the highest reliability. Meeting such constraints for emergency traffic in the presence of other periodic traffic with diverse QoS requirements is a great challenge.

Aiming to address the above-mentioned challenges, this paper proposes McMAC, a MAC protocol with multi-constrained QoS provisioning for diverse traffic in WBANs. We classify traffic types based on their multi-constrained QoS requirements and introduce a novel superframe structure based on the “transmit-whenever-appropriate” principle that allows diverse periods for diverse traffic classes based on their respective QoS demands. We exploit the advantages of both the contention-based and the contention-free approach that is best suited for the relevant traffic type. We adopt the receiver-driven poll-based data transmission mechanism for higher reliability and better channel utilization. We also devise a novel “emergency packet” handling mechanism that ensures packet delivery with the least possible delay and the highest reliability. Furthermore, we develop an analytical model for McMAC and perform extensive simulations to evaluate the performance of McMAC.

The rest of the paper is organized as follows. Section 2 summarizes the related work. Section 3 states some preliminaries behind our protocol design. Section 4 presents the detailed design of McMAC. Section 5 discusses the performance of our protocol using simulations. Finally, Section 6 provides our concluding remarks.

## 2. Related Works

To date, a number of potential contributions to WBAN QoS designs can be found in [18–25], notwithstanding the primary focus on energy-efficient MAC solutions. Most of these works adopt the IEEE 802.15.4 standard [26] or its variants to satisfy various QoS requirements for WBAN. In 802.15.4, a beacon-enabled or a non-beacon-enabled mode decides how to execute the medium access operation. All the existing proposals adopt the beacon-enabled mode to achieve reliable MAC control. In this mode, the superframe is divided into a beacon period, a contention access period (CAP), a contention-free period (CFP), and an inactive period. In the CAP, the nodes use the slotted CSMA/CA for slot reservation during CFP, which ensures guaranteed transmission. However, this standard does not have any traffic prioritization technique that could support diverse traffic types based on their QoS demands. Moreover, the limited number of guaranteed time slots may not be sufficient for WBAN applications.

PNP-MAC [18] prioritizes traffic based on emergency, medical, and non-medical types and extends IEEE 802.15.4 by introducing an advertisement period for basic network information, a number of beacon periods for providing the slot allocation status, a contention access period for non-periodic data and emergency alarm transmission, a number of data transmit slots (DTS), a number of emergency transmit slots (ETS), and an inactive period. PNP-MAC allows preemptive transmission during DTS for high-priority data and non-preemptive transmission during ETS, because emergency data are given the highest priority. Although PNP-MAC achieves a much better performance than 802.15.4 with regard to latency for higher-priority traffic, it does not differentiate among the QoS constraints for diverse traffic. In particular, some high priority traffic may require delivery with the least possible delay but can tolerate loss to some extent. Hence, treating traffic classes based on their priority might only waste resources.

Service differentiation based on traffic priority is also used in [21,22]. In [22], the authors mapped the application level traffic priorities into four access categories. The work assumes multiple queues at the MAC layer to support different access categories, which is not practical for WBAN nodes. An IEEE 802.11e-like approach was adopted to provide QoS for diverse traffic priorities. The authors in [21] proposed three traffic categories, namely, alarm/control (AC), command/data (CD), and routine (RT), and created a framing-structure-turning procedure to improve throughput, minimize queuing delay, and lessen energy consumption.

Zhang *et al.* [20] proposed a diversified CFP for two classes of traffic: periodic and bursty. They also divided the CAP into two control channels: AC1 and AC2. Although their work introduced the concept of diverse periods for diverse traffic classes, the classification was based only on the periodicity of the traffic instead of on diverse QoS demands. Khaled *et al.* [19] also classified traffic based on critical and non-critical issues. However, their work mainly concentrated on determining the number of retransmissions based on traffic criticality and avoided the other QoS issues.

Some proposals have promoted QoS provisioning issues in WBAN with different concerns. *RACOON* [24] is a QoS-aware MAC protocol that mainly focuses on multi-user QoS provisioning for inter-WBAN. BodyQoS [25], one of the primary QoS solutions for WBAN, separates the QoS scheduler from the underlying MAC protocols. *DQBAN* [23] exploits the fuzzy-logic system with multiple queues to improve the reliability of traffic.

Recently, a new task group IEEE 802.15.6 [27] has been established for the standardization of WBANs. In the beacon enabled mode of this standard, the superframe structure is divided into an exclusive access phase 1 (EAP1), a random access phase 1 (RAP1), two type I/II phases, an exclusive access phase 2 (EAP 2), a random access phase 2 (RAP 2), and a contention access phase (CAP). Nodes contend for the resource allocation during EAP, RAP and CAP periods, using either CSMA/CA or slotted Aloha access approach. The EAP1 and EAP2 provide the support for highest priority traffic such as reporting emergency events while the RAP1, RAP2, and CAP are used for regular traffic only. The Type I/II phases are used for uplink allocation intervals, downlink allocation intervals, bilink allocation intervals, and delay bilink allocation intervals. Unlike the IEEE 802.15.4, this standard provides diverse periods for diverse priority traffic classes. Nevertheless, treating each traffic class based on its multi-constrained QoS demand is ignored in this standard.

Apart from the state-of-the-art QoS-aware protocols, we introduce a novel QoS-aware solution for WBAN that is generic, energy-efficient, and guarantees multi-constrained QoS depending on the application requirement. This will be introduced in the following sections.

## 3. Preliminaries and Design Goals

### 3.1. System Models and Assumptions

**Figure 1.**
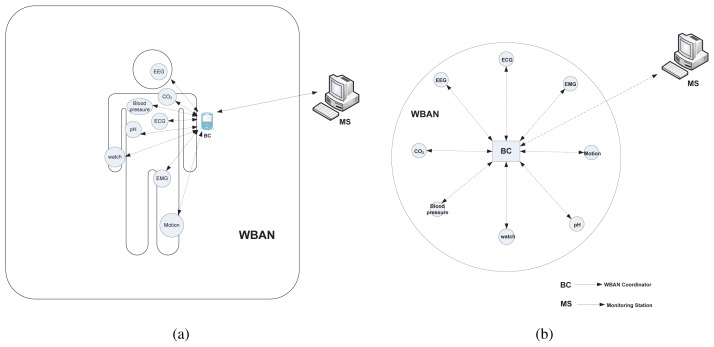
Network Model.

We consider a point-to-multipoint (star) network architecture as illustrated in Figure 1(b) for a WBAN setup with diverse types of WBAN nodes as depicted in Figure 1(a). One central node acts as a WBAN coordinator, denoted as *BC*, which is a full-function device (FFD). A set of WBAN nodes, denoted as ℕ*_BAN_*, either implanted or wearable, are reduced-function devices (RFD) that can communicate directly with the coordinator. Hence, the communication architecture between a WBAN node and the coordinator is single hop. As a FFD, the *BC* can perform some enhanced functionalities (*i.e.*, synchronization with the surrounding WBAN nodes, slot allocation, exchanging control packets, *etc.*); in contrast, as a RFD, a WBAN node performs only the sensing and transmitting of the sensed data to the *BC*. The WBAN nodes are usually battery-powered and hence energy-constrained. The *BC*, in contrast, is assumed to have an external power supply with higher processing capabilities than WBAN nodes. The *BC* processes the received data from the nodes and then sends it to the monitoring station or server through other networks (*i.e.*, cellular, WLAN, or wired); this communication paradigm is beyond the scope of this paper. There may also exist multiple *BC*s with different independent WBANs that are attached to the monitoring station. However, in this paper, our concern is to design a MAC framework within a single WBAN.

The WBAN nodes are categorized according to the type of traffic they generate, as presented in Section 3.2. Throughout this paper, the *i_th_* node generating traffic of type *j* ϵ 𝕋 is denoted as $n_{i}^{j}$, where T is the set of traffic class.

### 3.2. Traffic Classification

McMAC is mainly designed for the QoS provisioning of traffic with diverse requirements. Considering delay and reliability as the primary QoS constraints in the context of WBAN, we classify the traffic as follows:

- *Type 0*, Emergency traffic: This type of traffic possesses hard QoS constraints both in delay and reliability. It is usually event-triggered traffic and is generated whenever a life-critical situation occurs. For instance, when the heartbeat rate of a patient exceeds a certain threshold, an emergency action is needed, which thereby requires instantaneous transmission with the highest reliability.
- *Type 1*, Both delay and reliability constrained: The traffic belonging to this type has stringent delay and reliability requirements. However, compared with Type-0 traffic, it requires meeting the soft QoS rather than the hard QoS. A number of medical applications, for instance, electroencephalogram (EEG), electrocardiogram (ECG), and electromyography (EMG), *etc.*, generate real time medical continuous data that must be delivered with higher reliability within a certain deadline.
- *Type 2*, Reliability-constrained but not delay-constrained: This type of traffic has strict reliability requirements (nearly 100%) but can tolerate delay. An example of this type includes the transmission of medical images, such as X-ray, dermatology images, *etc.*, along with some vital sign monitoring applications, such as respiration monitoring, pH-level monitoring, *etc.* The traffic belonging to this application can be processed off-line and hence is not delay-constrained, but packet losses may cause disastrous consequences.
- *Type 3*, Delay-constrained but not reliability-constrained: This traffic type must meet a certain deadline; however, it can tolerate some packet losses. Examples of this type include telemedicine video streaming applications, recently developed consumer electronics application (*i.e.*, music for headsets), *etc.* Although the packet loss in this type of applications degrades the quality to some extent, the traffic validity is still preserved.
- *Type 4*, No constraint in either delay or reliability: This type of traffic does not have any strict delay or reliability constraints. The regular measurement of a patient's physiological parameters, such as temperature, pressure, *etc.*, corresponds to this class of traffic.

Notably, the traffic classification, in practical, is context-dependent. For instance, the QoS requirements of vital sign monitoring applications for a normal patient and for a patient in an emergency situation must be different. In the latter case the traffic corresponds to type 0, whereas in the former it may be categorized as type 2 or type 4. The traffic type for the sensors could be set a priori when attaching to the body or can be reset through special MAC command packets sent by the BC.

## 4. McMAC Design

### 4.1. Superframe Structure

**Figure 2.**
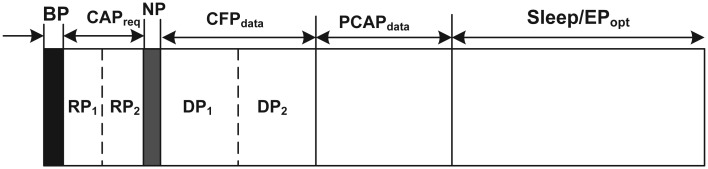
Superframe Structure.

To deal with traffic with heterogeneous QoS requirements, McMAC uses a superframe structure having distinct periods for diverse traffic, as illustrated in Figure 2. The main objective of such a structure is to “transmit-whenever-appropriate” such that nodes generating a particular type of traffic are allowed to transmit during the period that is best suited for meeting the corresponding QoS.

As depicted in Figure 2, the superframe starts with a Beacon Period (BP), where the BC broadcasts a beacon to the WBAN nodes that includes information for synchronization, structure parameters (*i.e.*, duration of BP, NP, *CAP_req_*, *CFP_data_*, *etc.*), and beacon load. Unlike the IEEE 802.15.4 standard, the beacon in McMAC is not used for allocating the time slots to the corresponding nodes during *CFP_data_*.

During the *CAP_req_*, nodes generating type 1 and type 2 traffic are allowed to send their slot allocation requests to the BC for the period *CFP_data_*. This period is further divided into two periods; a Request Period for type 1, denoted as *RP*_1_, and a Request Period for type 2, denoted as *RP*_2_. The rationale behind this is to reduce the contention probability significantly, because only a small set of nodes from ℕ*_BAN_* participate during their respective periods, which in turn increases the reliable reception of the request by the BC. More importantly, although the type 1 and type 2 traffic will be transmitted in a collision-free slot during *CFP_data_*, the prerequisite for maintaining this reliability is the reliable reception of the request packets of the reliability-constrained traffic.

The notification period, *NP* is used to send notification of the allocated slots from the BC to the nodes that sent the request during *CAP_req_*. A notification frame is used for such purpose. The use of NP thus facilitates the nodes generating delay-sensitive traffic, such as type 1 sending data within a single superframe without waiting for the next superframe, as is done in a typical 802.15.4 specification.

The period followed by NP is *CFP_data_*, where nodes having traffic with loss-intolerant properties (*i.e.*, type 1 and type 2 traffic) are allowed to transmit data in a reliable manner, because the nodes are given a contention-free slot, allocated by the BC, during NP. This period is further divided into two distinct periods: *DP*_1_ and *DP*_2_ for type 1 and type 2 traffic, respectively. However, the lengths of *DP*_1_ and *DP*_2_ are set dynamically based on the requests during *CAP_req_*. Due to the delay-sensitive nature of the type 1 traffic, the requests for those nodes are served first, and then the slots for the type 2 traffic are allocated.

McMAC allows the transmission of non-reliability-constrained traffic (both type 3 and type 4) during the prioritized contention access period for data, denoted as *PCAP_data_*. The prioritized contention access is used to give priority to delay-constrained type 3 traffic over non-delay-constrained type 4 traffic.

The last period of the McMAC superframe is the sleep or optional emergency period. During this period, nodes remain in sleep state, or nodes having emergency data (type 0) may transmit their data to the BC. Remarkably, the separate sleep period is not meant for all the nodes that must be in active state before this period, because each node turns its radio off immediately after finishing its transmission in its corresponding period. Hence, the sleep period here refers to all the nodes not carrying emergency traffic, which must be in the sleep state as all other periods for data transmission have already ended.

This “transmit-whenever-appropriate” superframe structure in McMAC provides the following advantages:

- We argue that providing contention-free slot allocation is much more resource-hungry than providing a contention access period because it requires a separate slot-request period, a notification period, and providing slot-allocation information through a beacon/notification frame. Because the main benefit of CFP in terms of QoS is ensuring higher reliability, McMAC allows only reliability-constrained traffic (type 1 and type 2) using this period with the aim of the best utilization of resources.
- The distinct CAP for sending the request packet and the data packet increases the reliability for both packet types as contention is reduced. For the same reason, we provide separate request periods for both type 1 and type 2 traffic during *CAP_req_*.
- The *PCAP_data_* in McMAC is used for non-reliability-constrained (type 3 and type 4) traffic. Type-3 traffic is usually bursty and has delay constraints. To transmit the packets with lower delay for the type 3 traffic, a prioritized medium access mechanism is used.
- Due to its delay-sensitive property, the type 1 traffic is prioritized over the type 2 traffic during *CFP_data_*. In particular, slots for the type 2 traffic are allocated only after all requests for the type 1 traffic have been satisfied.
- The exclusive periods for diverse traffic also yield benefits in energy savings because only the nodes with corresponding traffic remain active in their respective periods until their communication ends.
- McMAC does not use any separate period for type 0 traffic, because emergency traffic is usually event-triggered, may occur at any time, and needs to be transmitted instantaneously. The emergency traffic handling mechanism for different periods is discussed in detail in Section 4.3.

### 4.2. Medium Access Mechanisms

McMAC exploits diversified medium access mechanisms in diverse periods for better resource utilization, along with satisfying the traffic QoS demands, as presented below:

#### During *CAP_req_*

**Figure 3.**
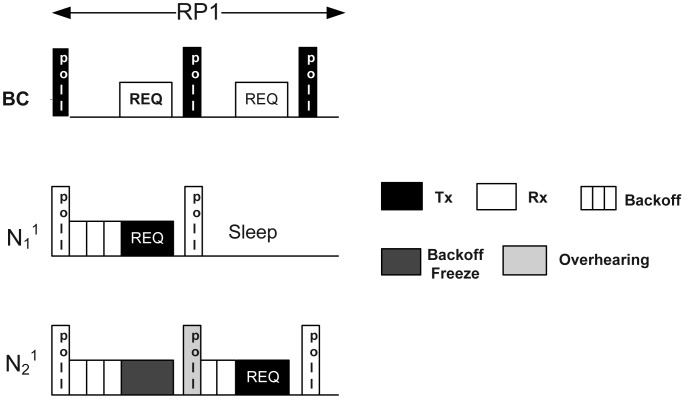
Medium access during *CAP_req_*.

McMAC uses polling-based medium access mechanisms during *CAP_req_*. Figure 3 illustrates the medium access mechanism during *CAP_req_*. The mechanism is shown only during RP1, because both RP1 and RP2 use the same mechanism.

As depicted in the figure, the BC broadcasts a poll packet during the first slot in RP1 and waits for a Timeout period equal to maximum backoff period before it receives a packet. The poll packet is a small packet that invites data transmission to the corresponding nodes. On receiving it, the respective nodes having the packet in their queue start counting down their random backoff within [1 ∷ 2*^n^* − 1]. Here, every node must do backoff for at least one backoff period, denoted as *aUnitBackoffPeriod*, to address the emergency traffic, as discussed later in Section 4.3. The winning node immediately transmits its request packet to the BC after the backoff period expires. The request packet contains the node *ID*, along with the number of data slots required during *CFP_data_*. Meanwhile, the losing nodes freeze their backoff counter on sensing that the channel is busy. After receiving the request packet from the winning node, the BC generates another poll packet in the next slot with an ACK bit set, which serves two purposes: it acts as an acknowledgment, and it solicits data packets from another node. The winning node goes into sleep state immediately after receiving the poll-ack. In contrast, the losing nodes resume their backoff on overhearing this poll-ack, and the procedure repeats until the corresponding period ends. If any collision occurs due to choosing the same random backoff by two nodes, the backoff procedure is repeated for a maximum number of times, *MaxNum_backoff_*, similar to the slotted CSMA/CA protocol. Notably, the BC continues sending the poll packet until the end of the respective period even if it does not receive any packet from the corresponding nodes after waiting for the maximum backoff period.

There are several reasons for using the polling-based medium access mechanism rather than the usual slotted CSMA/CA. First, this period is used for transmitting only short request packets that are periodic in nature and requires higher reliability. All transmissions from the corresponding nodes are synchronized by the poll packet reception. Due to the guided transmission by the BC using poll packets, the chances for packet collision are reduced, which increases the reliable reception of the request packets (packet collision occurs only if two nodes choose the same random backoff). Second, in this mechanism, randomized backoff is sufficient to avoid collisions from multiple transmitters; in addition, it does not need long CCA periods, which ensures better channel utilization. Finally, the backoff-freezing mechanism also reduces the unnecessary backoff counting that can occur when a new back-off procedure is started after every “channel busy” sensing.

#### During *CFP_data_*

**Figure 4.**
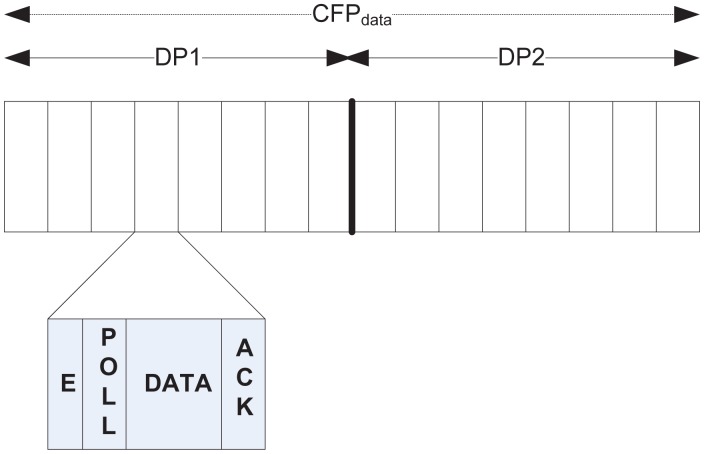
Slot Structure during *CFP_data_*.

As mentioned earlier, *CFP_data_* is used for collision-free data transfer for both type 1 and type 2 traffic during the sub-periods DP1 and DP2 respectively, where an exclusive slot is allocated for the nodes that sent a request during *CAP_req_*. On receiving notification about the allocated slot during NP, a node wakes up during its respective slot. Figure 4 illustrates the slot structure during *CFP_data_*. Both DP1 and DP2 follow the same slot structure, with every slot further divided into a number of mini-slots of variable length. The first mini-slot is for sending a short emergency tone, the use of which is discussed later in Section 4.3. This is followed by a polling slot, a data slot, and an ack slot. After it wakes up at the beginning of a slot, a node waits until the polling slot comes. During this slot, the BC sends a poll packet that invites data transmission. A node immediately transmits its data to the subsequent data slot, which is acknowledged by the BC during the ack slot.

#### During *PCAP_data_*

Similar to *CAP_req_*, *PCAP_data_* also exploits the polling-based medium access mechanism, where a node, on waking-up and having either type 3 or type 4 traffic, receives a poll packet from the BC in the very first slot of *PCAP_data_*. The nodes also start their random backoff just after receiving the poll packet. However, to prioritize type 3 over type 4 traffic, a random backoff period for a node *i* having *j*th traffic, denoted as ${\text{Backoff}}_{N_{i}^{j}}$, is chosen as follows:

$${\text{Backoff}}_{N_{i}^{j}}=\{\begin{array}{ll} 1∷2^{n}-1, & if(j=3)\\ 2^{n}∷2^{m}, & if(j=4) \end{array}$$

where, *n* and *m* are the backoff exponents for type 3 and type 4 traffic, respectively, and *n* < *m*. This diversified backoff period ensures that type 3 traffic will always be given higher priority for medium access in that superframe to address its delay sensitivity. However, the backoff freezing mechanism also prevents a node with type 4 traffic from suffering channel access starvation, when it waits for a long time. All other medium access operations during this period are similar to those in period during *CAP_req_*, as discussed earlier.

### 4.3. Emergency (Type-0) Packet Handling Mechanism

McMAC does not designate any separate period for emergency (type 0) packet due to two reasons: *First*, type 0 traffic is usually event-triggered and hence sporadic in nature. Thus, the allocation of a separate period causes unnecessary waste of resources in highly resource-constrained WBANs. *Second*, type 0 traffic is highly QoS-constrained in terms of both delay and reliability. The transmission of this traffic must be done instantaneously to satisfy the extensive delay-sensitive requirement. Hence, any delay incurred due to waiting for a separate period from the time the packet originated may invalidate the packet.

Therefore, McMAC handles the type 0 packet in a preemptive manner. In particular, regardless of the period in which this traffic is generated, McMAC preempts the corresponding traffic of that period. The handling mechanism of this traffic during different periods is discussed below:

#### During *CAP_req_* and *PCAP_data_*

**Figure 5.**
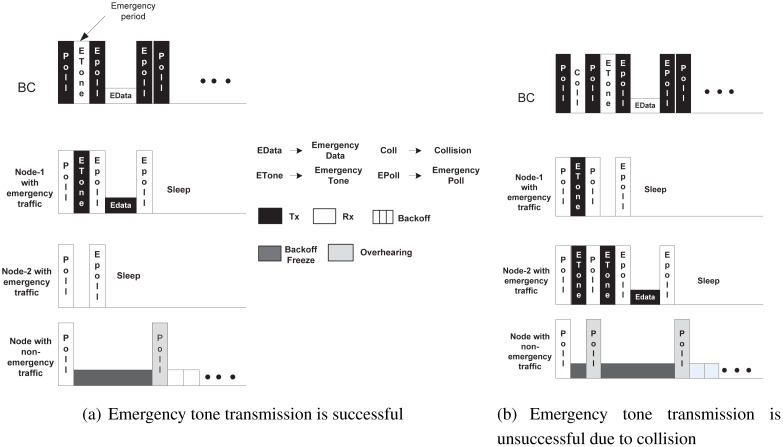
Emegency Packet handling during contention access periods.

McMAC handles the type 0 packet similarly in both contention access periods, *CAP_req_* and *PCAP_data_*. Because both of these periods are based on a polling-based medium access mechanism, nodes originating type 0 traffic during these periods wait until they receive a poll packet from the BC. Figure 5 illustrates this mechanism. A node broadcasts an emergency tone just after the immediate *aUnitBackoffPeriod* in which it received the poll packet, which we refer to as the emergency period. This period is expected to be free from transmission from any other nodes having traffic other than type 0, because the backoff needs to be performed for at least the *aUnitBackoffPeriod*.

There may exist multiple nodes with type 0 traffic waiting to broadcast an emergency tone in the same period. Hence, to address the contention, a node broadcasts the emergency tone with some probability *p* in that period. Two cases might happen in this scenario. First, if the transmission of the tone is successful (Figure 5(a)), the BC transmits a poll packet with emergency bit = 1 (which we refer to as an emergency-poll packet), along with the ID of the winning node in the subsequent period of the emergency period, which is followed by the transmission of emergency data by the winning node. The BC then acknowledges the reception of the emergency data with another emergency-poll packet with an ack bit set. The losing nodes with type 0 traffic readily go into sleep state after overhearing the ID in the emergency-poll packet. Second, if the transmission of the tone is unsuccessful (collision occurs with other tones) (Figure 5(b)), the BC transmits a regular poll packet (emergency bit = 0), and the losing nodes may attempt transmitting their tone again in this emergency period; if the nodes are successful, the data are transmitted following the same procedure described earlier.

All other nodes having traffic other than type 0 in their corresponding periods, which are already in the backoff state, immediately freeze their backoff on sensing that the channel is busy during the emergency period, and they will remain in that state until the transmission for the type 0 traffic ends. Those nodes resume their backoff as soon as they overhear a regular poll packet from the BC requesting regular data transmission.

#### During *CFP_data_*

As illustrated in Figure 4, the first mini-slot of a slot during *CFP_data_* is designated for sending an emergency tone. Hence, a node having type 0 traffic during that period broadcasts the emergency tone with probability *p*. The successful reception of the tone by the BC activates it, sending the poll packet with an emergency bit set, along with the ID of the winning node. This is followed by the exchange of emergency data and the respective ack in their corresponding mini-slots. However, if a collision occurs during the emergency mini-slot, the subsequent poll packet will be transmitted as a regular packet that invites the designated slot assignee node using that slot for data transmission, and the failure nodes with emergency traffic may keep trying to transmit the type 0 data in the next slot of *CFP_data_* until successful transmission is achieved.

The preemptive transmission of emergency traffic during a slot in *CFP_data_* necessitates the allocation of that slot to the node that it was earlier designated to. McMAC follows the following strategies in this case:

- If the preemption is done in a slot during *DP*1, the BC notifies the relocated slot information in the poll packet of the node in that preempted slot. This is important for a node in *DP*1 to satisfy its delay constraint. Although the transmission is delayed to some extent, it is guaranteed to be completed during that superframe. On receiving the new slot information, the node goes into sleep state immediately and then wakes up during the relocated slot. The BC usually chooses a slot in *DP*2 for the relocation of a particular slot in *DP*1, because the nodes in *DP*2 do not have any delay constraint.
- If the emergency traffic preempts a slot in *DP*2, no relocation for the slot is done, and the node(s) have to request for a slot again in the next superframe.

#### During Sleep/*EP_opt_*

As previously noted, during the Sleep/*EP_opt_*, the nodes remain in sleep state because all the communication for diverse traffic has already ended in the earlier periods. However, to take into account the occurrence of type 0 traffic, the BC continues sending the poll packet until the end of that superframe. Following the same mechanism, during *CAP_req_* and *PCAP_data_*, node(s) with type 0 traffic transmit the emergency tone just after the *aUnitBackoffPeriod* in which it received the poll packet with some probability *p*, and the same process continues for transmitting the type 0 packet.

Notably, because the BC totally controls the transmission of traffic of any type, it can dynamically determine whether it will allow preemption for type 0 traffic or not. This is sometimes important when the preemption decision is needed for type 1 traffic during both *RP*1 of *CAP_req_* and *DP*1 of *CFP_data_*. In some cases, it is sufficient to receive just one emergency packet to notify an emergency, because the other simultaneously generated packets may indicate the same situation. This also justifies using a separate, short emergency tone instead of sending a packet in the emergency slot to avoid wasting resource.

### 4.4. Analysis

#### 4.4.1. Analysis for Non-Emergency Traffic (Type 1 to Type 4)

We model the energy and delay performance for nodes having non-emergency traffic. We validate both the models with simulation results in Section 5. In this analysis, we assume *N* numbers of body sensor nodes having diverse periodic traffic (type 1 to type 4) transmitting packets to the BC. Because the BC is assumed to be non-energy-constrained and acts as a receiver of the data packets, we exclude it from this analysis. In this model, we assume that no emergency traffic is present and all the packets are transmitted successfully without any retransmission required.

##### A. *Energy analysis*

We focus on the radio energy consumption in body sensor nodes, as it is the most dominant source of energy consumption [28]. A radio device can be in one of the following states: listen, transmit, receive and sleep, which have different power consumption levels. Hence, the energy consumption of a radio device with *j*th traffic, denoted as *E^j^*^∈𝕋^, can be modeled by determining the fractional time it stays in each state per unit time (Equation (1)). We denote the power consumption in each state as *P_l_*,*P_t_*, *P_r_*, and *P_s_*, and the time spent in each state as $T_{l}^{j}$, $T_{t}^{j}$, $T_{r}^{j}$, and $T_{s}^{j}$ respectively.

(1) $$\begin{array}{cc} E^{j\in 𝕋} & =E_{t}+E_{r}+E_{l}+E_{s}\\ & =P_{t}T_{t}^{j}+P_{r}T_{r}^{j}+P_{l}T_{l}^{j}+P_{s}T_{s}^{j} \end{array}$$

Expected staying time during transmission:

(2) $$T_{t}^{j}=\underset{T_{t}^{j}(\text{RP}1/\text{RP}2)}{\underset{︸}{R_{d}^{j}L_{r}^{j}}}+\underset{T_{t}^{j}(\text{DP}1/\text{DP}2)}{\underset{︸}{R_{d}^{j}L_{d}^{j}}};\;\text{for}\;(j=1,2)$$

(3) $$T_{t}^{j}=\underset{T_{t}^{j}({\text{PCAP}}_{\text{data}})}{\underset{︸}{R_{d}^{j}L_{d}^{j}}};\;\text{for}\;(j=3,4)$$

Expected staying time during reception:

(4) $$T_{r}^{j}=\underset{\text{BP}+\text{NP}}{\underset{︸}{\frac{L_{b}+L_{n}}{d_{\text{sf}}}}}+\underset{T_{r}^{j}(\text{RP}1/\text{RP}2)}{\underset{︸}{R_{d}^{j}(2L_{p}+(K_{s}-1)L_{p})}}+\underset{T_{r}^{j}(\text{DP}1/\text{DP}2)}{\underset{︸}{R_{d}^{j}(L_{p}+L_{a})}};\;\text{for}\;(j=1,2)$$

(5) $$T_{r}^{j}=\underset{\text{BP}+\text{NP}}{\underset{︸}{\frac{L_{b}+L_{n}}{d_{\text{sf}}}}}+\underset{T_{r}^{j}({\text{PCAP}}_{\text{data}})}{\underset{︸}{R_{d}^{j}(2L_{p}+(K_{s}-1)L_{p})}};\;\text{for}\;(j=3,4)$$

Expected staying time during listening:

(6) $$T_{l}^{j}=\underset{T_{l}^{j}(\text{RP}1/\text{RP}2)}{\underset{︸}{R_{d}^{j}(T_{\text{bo}}+T_{f})}}+\underset{T_{l}^{j}(\text{DP}1/\text{DP}2)}{\underset{︸}{R_{d}^{j}T_{e}}};\;\text{for}\;(j=1,2),$$

(7) $$T_{l}^{j}=\underset{T_{l}^{j}({\text{PCAP}}_{\text{data}})}{\underset{︸}{R_{d}^{j}(T_{\text{bo}}+T_{f})}};\;\text{for}\;(j=3,4),$$

Expected staying time during sleeping:

(8) $$T_{s}^{j}=1-T_{t}^{j}-T_{r}^{j}-T_{l}^{j}$$

Equations (2)–(8) model the expected staying time of a node with *j*th traffic in each state. We use the symbols presented in Table 1 to model this expected staying time. Here, we assume that, all packets of *j*th type have a fixed length. All the packet sizes in the table are expressed in transmission time units. In Equations (2)–(7), the notation in the under-brace represents the expected staying time in the relevant periods as mentioned.

Equations (2)–(3) present the expected staying time during transmission, with Equation (2) modeling type 1 and type 2 traffic and Equation (3) exemplifying type 3 and type 4 traffic. In Equation (2), the expected transmission time includes the time spent during RP1 and DP1 (for type 1 traffic); and during RP2 and DP2 (for type 2 traffic). In contrast, for either type 3 or type 4 traffic, the expected transmission time includes only the time spent during *PCAP_data_*.

Equation (4) models the expected reception time for type 1 and type 2 traffic. It includes the beacon packet and notification packet reception in every superframe interval. Moreover, during the respective request period for type 1 and type 2 traffic, it includes the average poll packet reception time, which further depends on the expected number of successful attempts required for the node, denoted as *K_s_* to win the contention. In particular, if a node wins the contention during either RP1 or RP2 in its first attempt, then it receives at least two poll packets from the BC. However, an additional poll packet reception is required for every unsuccessful attempt. Here, a single attempt means starting/restarting the backoff timer after receiving a poll packet. During DP1 or DP2, it includes the reception of both the poll and ack packets in their corresponding slots. On the other hand, for type 3 and type 4 traffic, the expected reception time is similar to that during BP, NP, and RP1 or RP2 of Equation (4) as presented in Equation (5).

Equation (6) presents the expected listen time for type 1 and type 2 traffic. Here, the expected listen time during the respective request period includes the average backoff period and the average backoff freeze period, because nodes perform carrier sensing during both of these periods. The average backoff freeze period can be measured as:

(9) $$T_{f}=(K_{s}-1)(L_{r}+\text{SIFS})$$

which depends on the duration of the request packet transmission along with SIFS for every unsuccessful attempt to win the contention. During the data period for the corresponding traffic, the expected listen time includes only the emergency tone period. In contrast, as depicted in Equation (7), the average listen time for type 3 and type 4 traffic is similar to that during the request period in Equation (6).

Finally, the expected staying time during the sleep period is presented in Equation (8), which is formulated by measuring the staying periods for all other states.

##### B. *Delay analysis*

Here, we model the expected delay for delay-constrained traffic (type 1 and type 3). We assume that no delay is incurred due to retransmission and that the lengths of different periods in a superframe are sufficient to accommodate the corresponding generated traffic.

Delay modeling for type 1 traffic:

(10) $$D_{\text{worst}}^{1}=d_{sf}+\text{RP}2+\text{NP}+T_{N_{\text{slot}}}$$

(11) $$D_{\text{best}}^{1}=L_{p}+T_{\text{bo}}+L_{r}^{1}+\text{RP}2+\text{NP}+T_{N_{\text{slot}}}$$

(12) $$D_{\text{avg}}^{1}=\frac{d_{\text{sf}}}{2}+\text{RP}2+\text{NP}+T_{N_{\text{slot}}}$$

Equations (10)–(12) formulate the worst case, best case, and average case delay for type 1 traffic, denoted as $D_{\text{worst}}^{1}$, $D_{\text{best}}^{1}$ and $D_{\text{avg}}^{1}$ respectively. The worst case delay occurs when the traffic originates just after the RP1 of the corresponding superframe, the best case delay occurs when the traffic originates just before the poll packet transmission by the BC during RP1, and the average case delay occurs when the traffic originates at any time during a superframe. For each of these cases, every corresponding node suffers a fixed delay of RP2, NP, and *T_Nslot_*, where *T_Nslot_* denotes the time required for an allocated data slot to finish from the start of the *CFP_data_*. In addition, the worst case delay includes a delay of a complete superframe duration, *d_sf_*. The best case delay includes a poll packet reception time, an average backoff duration, and a request packet transmission time. The average case delay includes an average superframe duration, which is $\frac{d_{\text{sf}}}{2}$.

Delay modeling for type 3 traffic:

(13) $$D_{\text{worst}}^{2}=(d_{\text{sf}}-T_{{\text{PCAP}}_{\text{data}}})+T_{w}+L_{p}+T_{\text{bo}}+L_{d^{i}}^{3}+(K_{s}-1)(L_{p}+L_{d^{j\neq i}}^{3})$$

(14) $$D_{\text{best}}^{3}=L_{p}+T_{\text{bo}}+L_{d^{i}}^{3}$$

(15) $$D_{\text{avg}}^{3}=\frac{\left(d_{\text{sf}}-T_{{\text{PCAP}}_{\text{data}}}\right)}{2}+T_{w}+L_{p}+T_{\text{bo}}+L_{d^{i}}^{3}+(K_{s}-1)(L_{p}+L_{d^{j\neq i}}^{3})$$

The delay modeling for type 3 traffic is presented in Equations (13)–(15). The worst case delay for type 3 traffic of node *i* occurs when the traffic is generated just after the *PCAP_data_* ends. It includes: the duration until the next *PCAP_data_* comes, an average waiting time until the poll packet is received (denoted as *T_w_*), a poll packet reception time, an average backoff duration, the data packet transmission time for node *i*, and the data packet reception time for all nodes *j* other than *i* for every unsuccessful attempt to win the contention. In contrast, the best case delay includes only the poll packet reception time, an average backoff period, and the data packet transmission time for node *i*, because such delay occurs when the traffic is generated just before the poll packet is received and the contention is won with a single attempt. Because the average case delay considers the packet generation at any time during the superframe, it takes on the average duration of $\frac{\left(d_{\text{sf}}-T_{{\text{PCAP}}_{\text{data}}}\right)}{2}$, and the remaining periods carry the same meaning as described for the worst case delay.

#### 4.4.2. Analysis for Emergency Traffic (Type 0)

In this analysis, we model the reliability and expected delay for emergency traffic in McMAC.

##### A. Reliability Modeling

Let *P_e_* be the probability of successful contention in a single emergency time slot and *n_e_* be the number of nodes present having emergency traffic during a particular period. Hence, *P_e_* can be formulated as

(16) $$P_{e}={\left(1-\frac{1}{n_{e}}\right)}^{n_{e}-1}$$

Assuming the maximum number of attempts for successful transmission is *K*, the probability of successful transmission, denoted as *P_succ_*, can be derived as:

(17) $$P_{\text{succ}}=\sum_{i=1}^{K}P_{e}{\left(1-P_{e}\right)}^{i-1}$$

From Equation (17), we can also determine the number of emergency access slots needed to have a certain degree of reliability. Table 2 illustrates the number of emergency slots required for different packet success rates assuming *n_e_* = 3.

##### B. Delay Modeling

The expected delay for emergency traffic during the contention access period (*CAP_req_*/*PCAP_data_*) can be formulated as

(18) $$D_{\text{CAP}}^{E}=T_{w}^{\text{CAP}}+(K-1)(L_{\text{et}}+L_{p})+L_{\text{et}}+3L_{p}+L_{\text{ed}}$$

where $T_{w}^{\text{CAP}}$ denotes the average waiting period until a node receives a poll packet during CAP, *K* is the number of attempts required to successfully transmit the emergency packet, *L_et_* is the emergency tone reception time, and *L_ed_* denotes the emergency data packet transmission time. Because the structure of the regular poll packet is the same as that of the emergency poll packet, we use the same notation *L_p_* for both packets in this equation.

We formulate the expected delay of the emergency packet during the contention free period as

(19) $$D_{\text{CFP}}^{E}=T_{w}^{\text{CFP}}+K.T_{s}^{\text{CFP}}$$

where $T_{w}^{\text{CFP}}$ is the average waiting period until a node receives a poll packet during *CFP*, and $T_{s}^{\text{CFP}}$ denotes the duration of a time slot during *CFP*. Because for every unsuccessful transmission of emergency data during *CFP* every node must wait for the next time slot, a node has to suffer a delay of $K\times T_{s}^{\text{CFP}}$ for the successful transmission of an emergency packet.

## 5. Performance Evaluation

This section presents the performance evaluation of the McMAC protocol. The results illustrate that McMAC successfully achieves its design goals.

### 5.1. Simulation Environment

We consider a single WBAN consisting of a single BC and several WBAN nodes with diverse traffic. The network topology is single-hop star-type (point-to-multipoint), as illustrated in Figure 1 in Section 3.1. Considering the normal application scenario of a WBAN, where all data transmissions are initiated by the WBAN nodes, we avoid the download traffic from the BC in this simulation. The simulation is performed using the ns-2 simulator version 2.34 with the IEEE 802.15.4 WPAN simulation extension from Zheng [29] of Samsung/CUNY.

Table 3 shows the different network parameters used in our simulation. In addition, the radio parameter values are taken from Table 1. During the simulation, we neglect the effect of bit errors in the channel and the interference from neighboring WBANs. In particular, a packet is dropped only due to packet collision or buffer overflow. Each node generates periodic data flow except for the emergency traffic, which occurs randomly. Here, we randomize the initial data generation of the nodes. In this study, we compare McMAC with the legacy IEEE 802.15.4 and PNP-MAC. Because PNP-MAC uses traffic prioritization, we map the priority of our proposed traffic classes in the following order: type 0−> priority 0, type 1−>priority 1, type 2−>priority 1, type 3−>priority 3, and type 4−>priority 2. Each simulation is performed for 1,000 seconds, and we average the values obtained in 30 random runs. In all the simulation results, error bars show 95% confidence interval.

### 5.2. Performance Metrics

In this study, we used the following metrics to evaluate McMAC.

#### Energy Consumption

The total energy consumption, *E*, is calculated as

(20) $$E=\sum_{i=1}^{n}P_{l}T_{l}^{i}+P_{t}T_{t}^{i}+P_{r}T_{r}^{i}+P_{s}T_{s}^{i}$$

where *n* is the number of deployed nodes. The average value of *E* is taken after 30 simulation runs.

#### Energy Efficiency

The energy efficiency metric [30,31], *E_f_*, is defined as

(21) $$E_{f}=\frac{\text{total amount of useful data delivered}\;(\text{bits})}{\text{total energy consumed}\;(\text{joule})}$$

This metric provides useful information regarding the effect of energy consumption on system throughput.

#### End-to-End Latency

The end-to-end latency of a packet is measured as the time difference between the packet generation time and the time when the acknowledgment of the packet is received from the BC. The delays experienced by distinct data packets are averaged over the total number of distinct packets received by the BC.

#### Reliability

The reliability is the ratio of the total number of unique packets received by the BC to the total number of packets generated by the WBAN nodes.

### 5.3. Simulation Results

This section presents the results obtained through evaluating McMAC using the metrics stated above. We evaluate different metrics, varying the number of WBAN nodes. Table 4 presents the traffic class distribution (except for emergency traffic) per superframe length for different numbers of WBAN nodes.

#### 5.3.1. Delay Performance

In this study, we evaluate the average latency of delay-constrained periodic traffic (type 1 and type 3) with or without emergency traffic.

**Figure 6.**
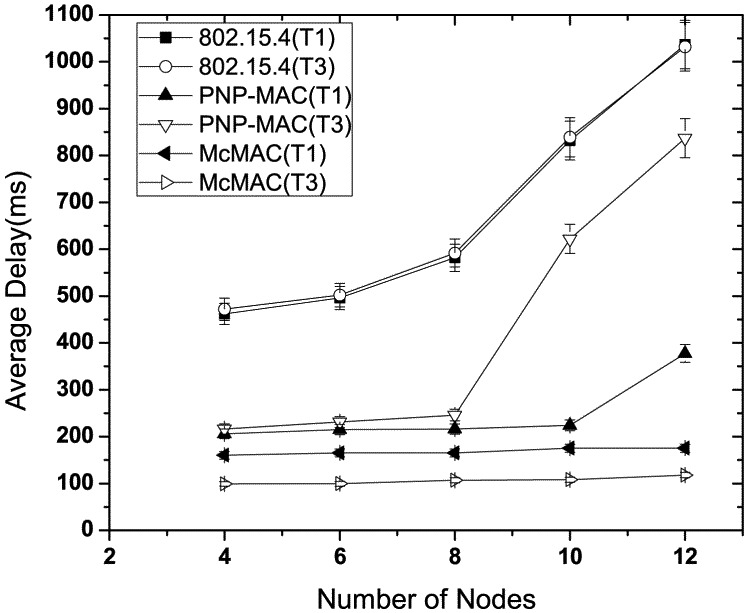
Average latency at varying number of nodes without emergency traffic.

Figure 6 illustrates the latency performance in the absence of any emergency traffic. As the network size increases, 802.15.4 exhibits the highest latency due to the allocation of GTS in the next superframe, along with limited number of GTS slots. Moreover, because all types of traffic are handled equally, both type 1 and type 3 show almost similar delay performances. In contrast, due to the prioritization of certain traffic types, PNP-MAC shows a much lower delay for both of these traffic cases. However, because type 3 traffic has the lowest priority in PNP-MAC, the end-to-end delay increases sharply when the network size exceeds the length of CAP. Among the other protocols, McMAC exhibits the best delay performance for both type 1 and type 3 traffic due to the allocation of diverse periods for diverse traffic. Comparatively, type 3 traffic shows a lower delay than type 1 in McMAC because it does not require an additional contention-free slot, which is needed for guaranteed transmission for type 1 traffic.

**Figure 7.**
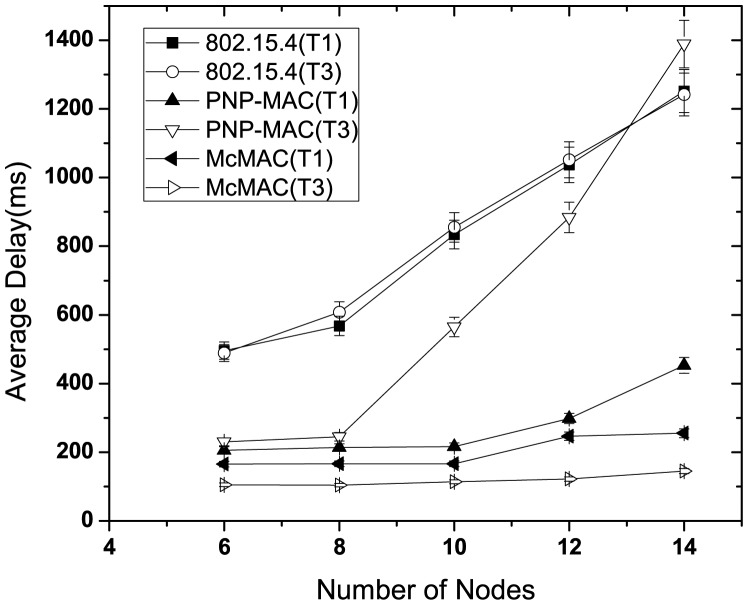
Average latency at varying number of nodes with emergency traffic.

Figure 7 illustrates the average delay performance of type 1 and type 3 traffic in the presence of nodes with sporadic emergency traffic. In this study, we use two additional nodes with emergency traffic, along with the network size and traffic distribution, as shown in Table 4. These two nodes generate traffic randomly for about 10 s until the simulation starts and until 80 packets in total are generated. This study shows the delay performance for this 10 s simulation time.

As depicted in Figure 7, in the presence of emergency traffic, the average latency increases almost linearly as the number of nodes increases for each traffic case of 802.15.4. Because the type 3 traffic has the lowest priority, the emergency traffic causes the worst delay for the type 3 traffic in PNP-MAC when the network size exceeds the CAP limit, which inhibits gaining channel access during the CAP. In contrast, because the type 1 traffic has higher priority, it is not affected as significantly by the emergency traffic. The average delay of type 1 traffic increases slightly with emergency traffic in McMAC as it shares the same slot (either during *CAP_req_* or *CFP_data_*) if the emergency traffic generation coincides with the type 1 traffic. However, emergency traffic has a negligible effect on type 3 traffic in McMAC as it exploits only *PCAP_data_* for its transmission.

**Figure 8.**
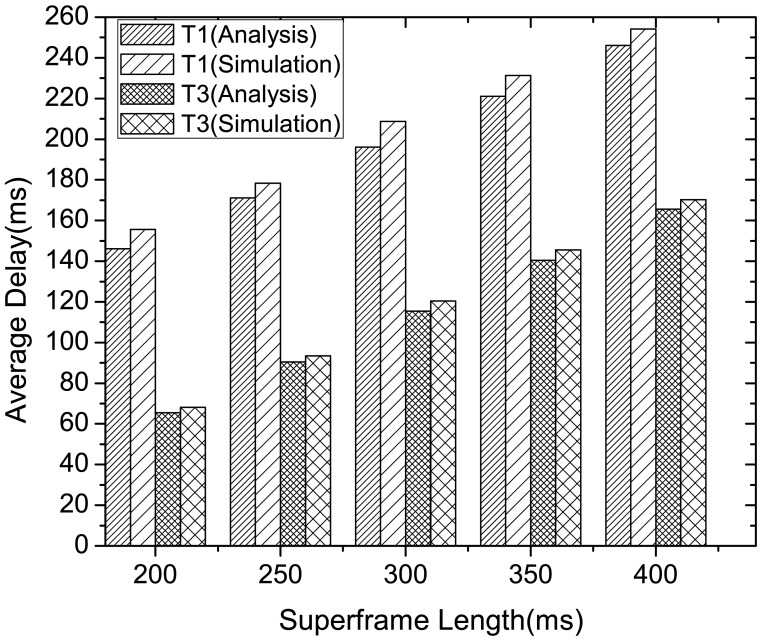
Delay performance comparison of type 1 and type 3 traffic both in analysis and simulation.

Figure 8 compares the analysis and simulation results of the latency performance of delay-constrained (type 1 and type 3) traffic in McMAC, varying the superframe length. The analytical results are evaluated using the values shown in Tables 1 and 3. Here, we consider *N* = 8 with two nodes from each traffic type, and each node generates four packets per second. As the figure illustrates, the latency performance in different superframe length are similar in both analysis and simulation results, where the average case delay increases with the increase in superframe length for both the traffic types. This signifies that an appropriate superframe length might be determined based on the application delay requirements for delay constrained traffic. Moreover, the type 3 traffic exhibits much lower latency than type 1 traffic as validated in both analysis and simulation. This is because the transmission of type 1 traffic requires waiting for an additional request period and notification period for contention-free transmission. Hence, in contrast to the case of type 3 traffic, the type 1 traffic can achieve higher reliability by compromising some latency.

#### 5.3.2. Reliability Performance

In this study, we evaluate the reliability performance of reliability-constrained traffic (type 1 and type 2) with or without emergency traffic. In evaluating the performance without emergency traffic, we followed the same set-up as discussed in the previous section.

**Figure 9.**
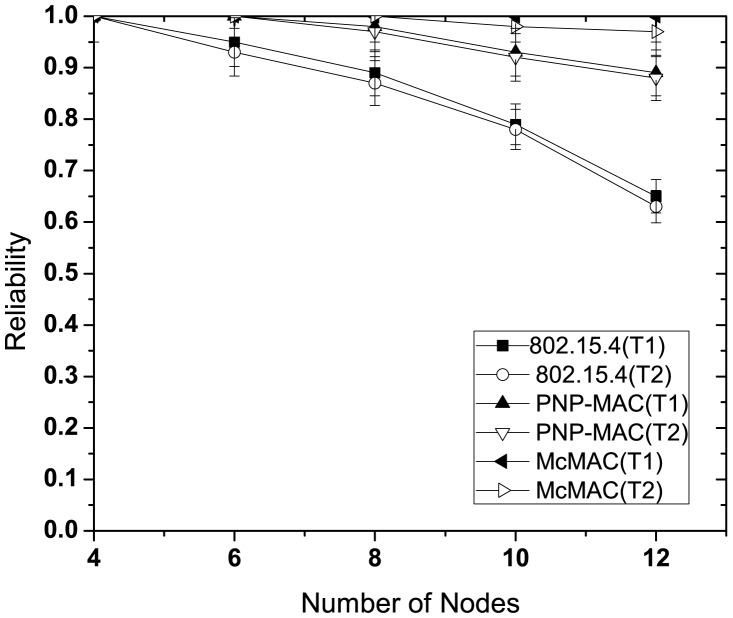
Reliability at varying number of nodes without emergency traffic.

Figure 9 illustrates the reliability for different number of nodes without emergency traffic. With the increasing number of nodes, the reliability decreases sharply for each traffic case of 802.15.4 due to increased collisions as the traffic load increases. Because of the higher priority assigned to both of these traffic types, PNP-MAC exhibits a much better reliability performance than 802.15.4. In contrast, we observe almost 100% reliability for both type 1 and type 2 traffic in McMAC due to the allocation of diversified periods for request packet transmission (which reduces contention to a greater extent), along with contention-free slot allocation.

**Figure 10.**
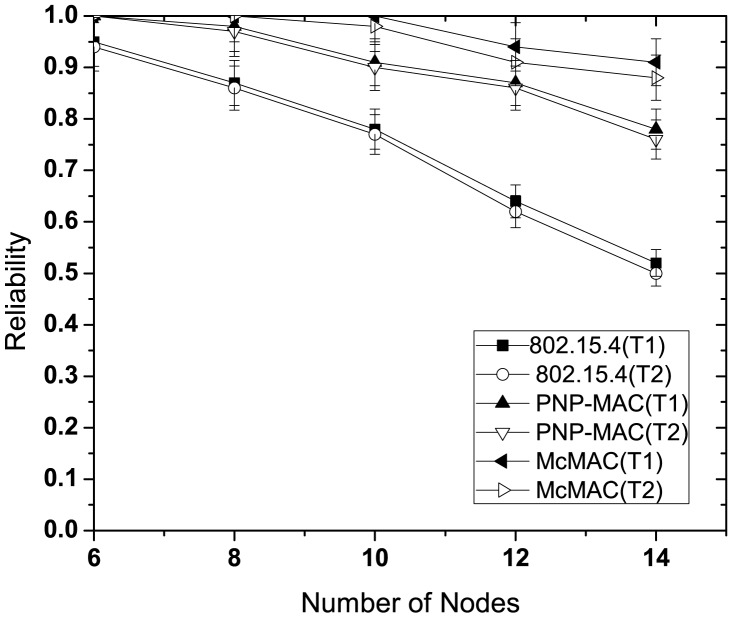
Reliability at varying number of nodes with emergency traffic.

The presence of emergency traffic decreases the reliability of type 1 and type 2 traffic for every case, as depicted in Figure 10. This is due to the limited CAP duration, which increases the contention with the additional emergency traffic. However, in spite of the separate CAP allocation for both type 1 and type 2 traffic in McMAC, the reliability decreases due to the slot sharing of emergency traffic with the periodic type 1 and type 2 traffic.

#### 5.3.3. Energy Performance

This study evaluates the average energy consumption of the nodes for different protocols. We also evaluate the average energy consumption for diverse periodic traffic in McMAC.

**Figure 11.**
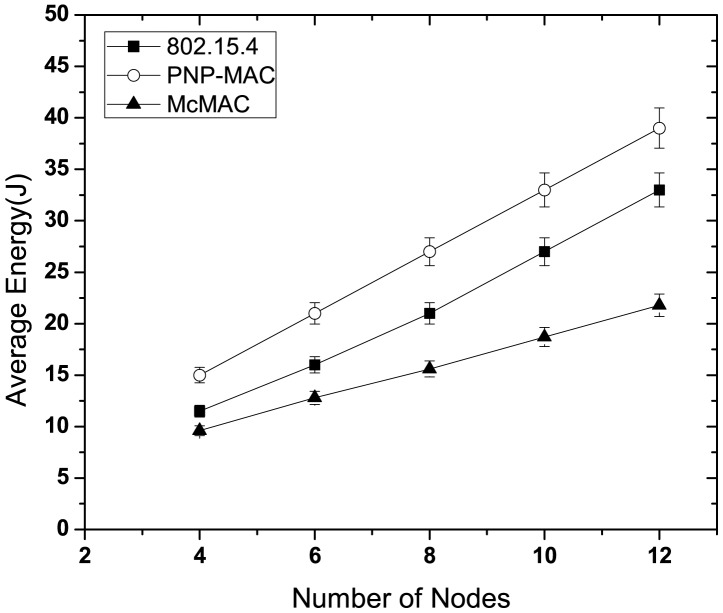
Average energy consumption at varying number of nodes.

Figure 11 illustrates the average energy consumption of the nodes for different network sizes. PNP-MAC exhibits the highest energy consumption due to its limited inactive period and the provisioning of two or more beacon periods, along with one advertisement period. Although McMAC possesses the same duration of inactive period as PNP-MAC, its energy consumption is the lowest, because not all the nodes remain active in the active periods. Due to the distinct allocation of periods, only the relevant nodes remain awake, and these enter sleep state after either the data exchange or the corresponding period ends, which considerably reduces idle listening and overhearing. With a 50% duty cycle, 802.15.4 also shows a higher energy consumption than McMAC because it has a long CAP period, which keeps the nodes awake.

**Figure 12.**
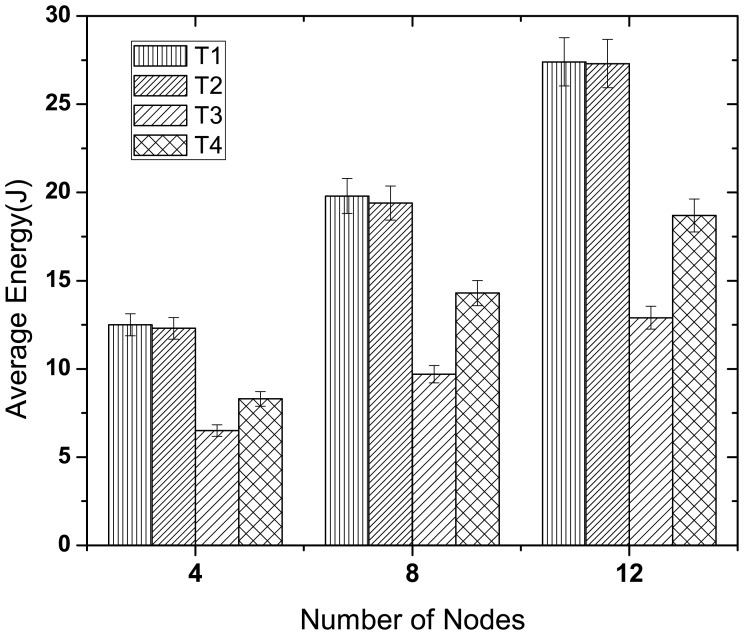
Average energy consumption of diverse periodic traffic at varying network size.

Figure 12 illustrates the energy consumption of all the periodic traffic types in McMAC. Here, we consider three network sizes (4, 8 and 12) with an equal number of traffic types in each case. As the figure shows, the type 1 and type 2 traffic consume almost similar amounts of energy for different network sizes, and their energy consumption is higher than that of type 3 and type 4. This is because two distinct periods (*CAP_req_* and *CFP_data_*) are required for guaranteed transmission for these reliability-constrained traffic (type 1 and type 2), whereas a single period (*PCAP_data_*) is needed for type 3 and type 4 traffic. However, type 3 traffic has to perform less backoff than type 4 because of its higher priority, which results in less overhearing and idle listening and hence lower energy consumption.

**Figure 13.**
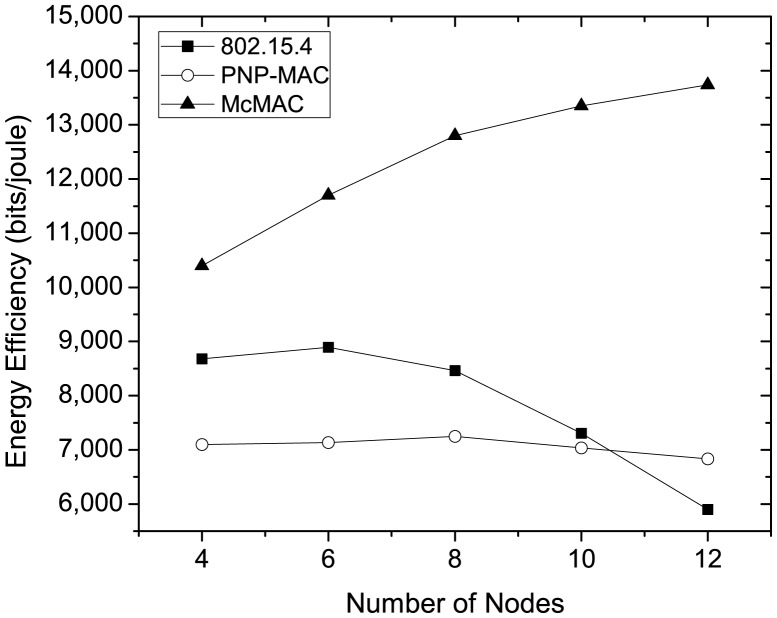
Energy efficiency at varying number of nodes.

Figure 13 shows the energy-efficiency for different protocols at varying traffic loads as the number of nodes increases. As the figure illustrates, PNP-MAC exhibits a lower energy-efficiency, and the efficiency decreases slightly as the traffic load increases. Although 802.15.4 has better energy-efficiency than PNP-MAC during low traffic loads, a sharp decrease is observed when the traffic load reaches a certain level (*N* = 8), and the energy-efficiency has the lowest value when *N* = 12. This is because the network throughput decreases dramatically for 802.15.4 at higher traffic loads, although it has better energy consumption than PNP-MAC. In contrast, McMAC shows the highest energy-efficiency for different traffic loads, as it has the lowest energy consumption and higher throughput, due to the diverse period allocation for diverse traffic types.

**Figure 14.**
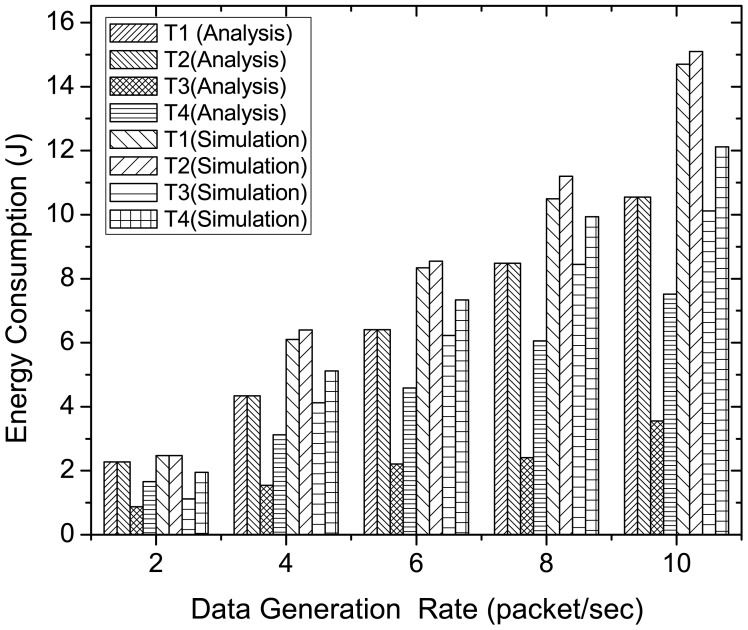
Comparison of energy consumption of diverse traffic types in analysis and simulation for different data generation rate.

Figure 14 compares the analytical and simulation results of energy consumption for diverse traffic in McMAC, varying traffic load in terms of data generation rate. Here, we consider *N* = 8 and the traffic distribution follows as shown in Table 4. As the figure shows, during low traffic rate (2 packets per second), both analytical and simulation results show similar energy consumption for all traffic types. In contrast, as the data generation rate increases, we observe higher energy consumption for the diverse traffic in the simulation results than that of analysis. This is because the energy model assumes an ideal environment with no packet loss. However, the energy expenditure due to retransmission is well reflected in simulation results at higher traffic rate. Besides, the simulation results validate the analysis results in terms of the ratio of the energy consumption for diverse traffic types.

#### 5.3.4. Performance of Emergency Traffic

In this study, we evaluate the performance of emergency traffic in terms of delay and reliability for different protocols. We adopt a similar network set-up to that described in Section 5.3.1. while evaluating the performance with emergency traffic.

**Figure 15.**
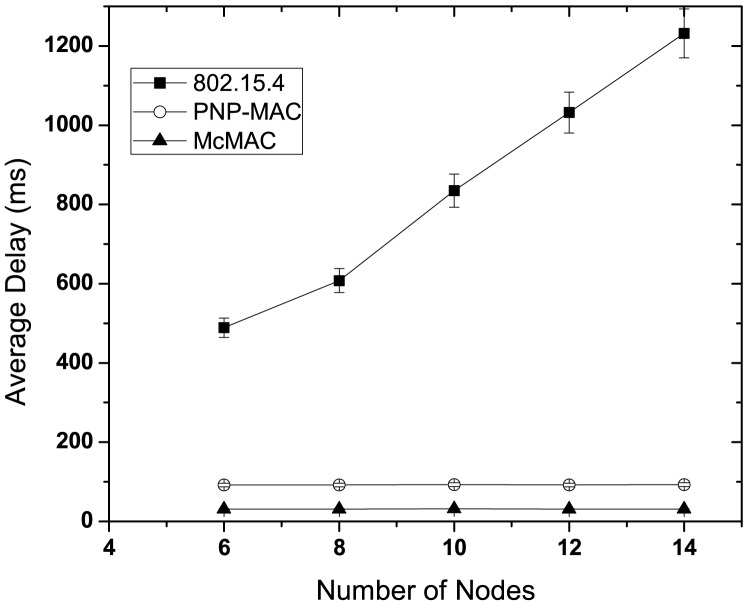
Average latency of emergency packet at varying number of nodes.

Figure 15 shows the average latency of emergency traffic for different numbers of nodes. Due to the special handling of an emergency packet, both PNP-MAC and McMAC exhibit a significantly lower delay than 802.15.4. However, McMAC uses the slotted-aloha like approach for emergency packet transmission, and it allows all the periods, except BP and NP, for emergency data. This causes a lower delay than that in PNP-MAC, where only CAP and ETS are allowed for emergency traffic. Moreover, the network size does not have any effect on the emergency packet in these two protocols, because it is given the highest priority in PNP-MAC and a separate emergency period is assigned after every poll packet transmission in McMAC.

**Figure 16.**
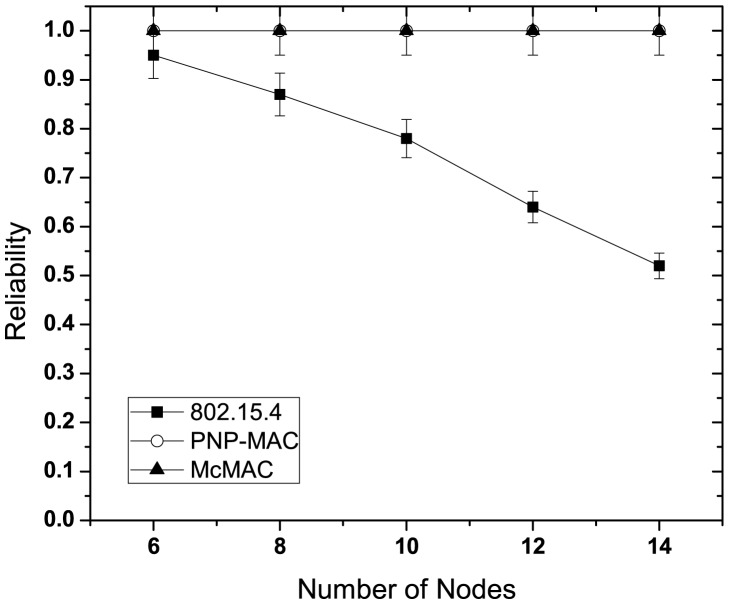
Reliability of emergency packet at varying network size.

Figure 16 illustrates the reliability of the emergency packet for different network sizes. Because of their respective emergency handling mechanisms, both PNP-MAC and McMAC achieve 100% reliability, whereas 802.15.4 shows a similar reliability performance to that of the non-emergency packet for different numbers of nodes.

**Figure 17.**
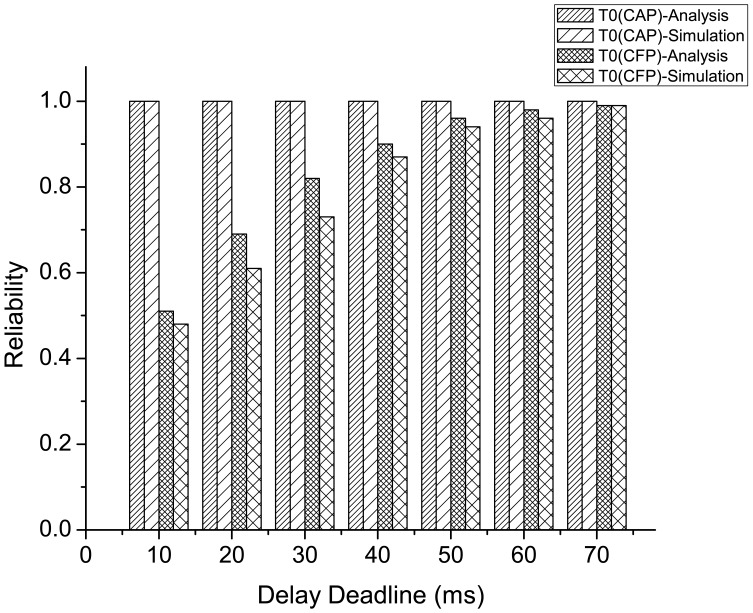
Comparison of analytical and simulation results on reliability performance of emergency traffic for different delay deadline.

Figure 17 compares the analytical and simulation results on the reliability performance of emergency traffic (both in CAP and CFP) for different delay deadline. The analytical results are evaluated using both the reliability and delay model of emergency traffic as presented in Section 4.4.2.. Here, the average delay is set to different delay deadline values, and the corresponding reliability is measured using the models. In this result, we consider *n_e_* = 3 and *N* = 8 with traffic distribution presented in Table 4.

As the figure shows, the analysis and simulation results exhibit similar performance on reliability at varying delay deadline for the emergency traffic originated during both CAP and CFP, where the reliability increases with a higher delay deadline. The increase in delay deadline for emergency traffic originated during CAP has negligible effect on the achieved reliability, since the occurrence of collision of emergency tone during an emergency slot delays the next transmission only for the next emergency slot instead of next data slot, which ensures higher reliability without incurring much delay. In contrast, the delay deadline has significant effect on the reliability performance for the emergency traffic originated during CFP. This is because the occurrence of collision during the emergency tone transmission in an emergency mini-slot of *CFP_data_* delays the transmission of the emergency tone to the subsequent data slot of *CFP_data_*, thus increasing the delay to a greater extent than that of the traffic originated during CAP.

## 6. Concluding Remarks

This paper presents McMAC, a MAC protocol with multi-constrained QoS provisioning for diverse traffic in WBANs. Concerning the diverse QoS requirements of heterogeneous traffic, a novel superframe structure is proposed that allows a node with a particular traffic type to transmit during the period that is best suited for meeting its corresponding QoS. To satisfy the QoS requirement of emergency traffic, which occurs sporadically, an emergency traffic handling mechanism is also presented.

The performance of McMAC was compared with that of PNP-MAC (a recent QoS-aware MAC protocol for WBAN) and IEEE 802.15.4 (a baseline protocol for WBAN) through extensive simulations. The comparison results demonstrate that McMAC successfully meets the delay and reliability requirements of diverse traffic types while keeping a low energy consumption.

## Figures and Tables

**Table 1.** Typical symbols and values used in McMAC radio energy and delay analysis.

Symbol	Meaning	Values

*P_l_*	Power in Listening	41.4 mW
*P_t_*	Power in Transmitting	36.5 mW
*P_r_*	Power in Receiving	41.4 mW
*P_s_*	Power in Sleeping	42 *μ*W

$R_{d}^{j}$	Data packet rate of *j*th traffic	varying
$L_{r}^{j}$	Request packet length of *j*th traffic	352 *μs*
$L_{d}^{j}$	Data packet length of *j*th traffic	80 *μs*
*L_b_*	Beacon packet length	48 *μs*
*L_n_*	Notification packet length	variable
*L_p_*	Poll packet length	32 *μs*
*L_a_*	Acknowledgement packet length during *CFP*	48 *μs*

*d_sf_*	Superframe interval	245.76 ms
$T_{bo}^{j}\in 1,2$	Average backoff period for traffic type 1,2	5.12 *ms*
$T_{bo}^{j}\in 3$	Average backoff period for traffic type 3	1.28 *ms*
$T_{bo}^{j}\in 4$	Average backoff period for traffic type 4	3.68 *ms*
*T_f_*	Average backoff freeze period	0.54 *ms*
*T_e_*	Duration of emergency slot	12 *μs*

**Table 2.** Number of emergency slots for different reliability.

Reliability (%)	No. of emergency slots(*K*)

69	2
82	3
90	4
95	5
97	6
98	7
99	8

**Table 3.** Parameters and their values used in the simulation.

Parameter	Value	Parameter	Value	Parameter	Value

ChannelRate	250 kbps	*BeaconLength*_802.15.4_	20 octets	SuperframeLength	245.76 ms
*Slotduration*	480 sym	*BeaconLength_McMAC_*	12 octets	*BO*_802.15.4_	4
SIFS	192 *μ* s	BufferSpace	1, 000 octets	*SO*_802.15.4_	3
*aUnitBackoffperiod*	320 *μ*s	PayloadLength	20 octets	*BP*	1 slot
Symbol Time	16 *μ*s	NotifyPacketLength	variable	*NP*	1 slot
PHYHeader	6 octets	PollPacketLength	8 octets	*CAP_req_*	6 slots
minBE	3	ReqPacketLength	11 octets	*CFP_data_*	10 slots
maxBE	5	EToneLength	3 octets	*PCAP_data_*	10 slots
*DTS_PNP_*	20 slots	*ETS_PNP_*	5slots	*CAP*_802.15.4_*_/PNP_*	8 slots

**Table 4.** Traffic class distribution varying network size.

No. of Nodes	Distribution of traffic class			
	Type 1	Type 2	Type 3	Type 4

4	1	1	1	1
6	2	2	1	1
8	2	2	2	2
10	3	3	2	2
12	3	3	3	3
